# Biotic and abiotic drivers of intraspecific trait variation within plant populations of three herbaceous plant species along a latitudinal gradient

**DOI:** 10.1186/s12898-017-0151-y

**Published:** 2017-12-12

**Authors:** Kenny Helsen, Kamal P. Acharya, Jörg Brunet, Sara A. O. Cousins, Guillaume Decocq, Martin Hermy, Annette Kolb, Isgard H. Lemke, Jonathan Lenoir, Jan Plue, Kris Verheyen, Pieter De Frenne, Bente J. Graae

**Affiliations:** 1 Department of Biology, Norwegian University of Science and Technology, Høgskoleringen 5, 7034 Trondheim, Norway; 2 Southern Swedish Forest Research Centre, Swedish University of Agricultural Sciences, Box 49, 230 53 Alnarp, Sweden; 3 Department of Physical Geography and Quaternary Geology, Stockholm University, 106 91 Stockholm, Sweden; 4 Edysan (FRE 3498 CNRS), Centre National de la Recherche Scientifique/Université de Picardie Jules Verne, 1 rue des Louvels, 80037 Amiens Cedex, France; 5 Division Forest, Nature and Landscape Research, Department Earth and Environmental Sciences, University of Leuven, Celestijnenlaan 200E, 3001 Heverlee, Belgium; 6 Vegetation Ecology and Conservation Biology, Institute of Ecology, FB 02, University of Bremen, Leobener Strasse 5, 28359 Bremen, Germany; 7 Forest & Nature Lab, Ghent University, Geraardsbergsesteenweg 267, 9090 Gontrode-Melle, Belgium; 8 Department of Plant Production, Ghent University, Proefhoevestraat 22, 9090 Melle, Belgium

**Keywords:** *Anemone nemorosa*, Between-individual ITV, Herbaceous plant species, *Impatiens glandulifera*, Individual variation hypothesis, Intraspecific trait variation, Latitudinal gradient, *Milium effusum*, Niche packing, Phenotypic plasticity

## Abstract

**Background:**

The importance of intraspecific trait variation (ITV) is increasingly acknowledged among plant ecologists. However, our understanding of what drives ITV between individual plants (ITV_BI_) at the population level is still limited. Contrasting theoretical hypotheses state that ITV_BI_ can be either suppressed (stress-reduced plasticity hypothesis) or enhanced (stress-induced variability hypothesis) under high abiotic stress. Similarly, other hypotheses predict either suppressed (niche packing hypothesis) or enhanced ITV_BI_ (individual variation hypothesis) under high niche packing in species rich communities. In this study we assess the relative effects of both abiotic and biotic niche effects on ITV_BI_ of four functional traits (leaf area, specific leaf area, plant height and seed mass), for three herbaceous plant species across a 2300 km long gradient in Europe. The study species were the slow colonizing *Anemone nemorosa*, a species with intermediate colonization rates, *Milium effusum*, and the fast colonizing, non-native *Impatiens glandulifera*.

**Results:**

Climatic stress consistently increased ITV_BI_ across species and traits. Soil nutrient stress, on the other hand, reduced ITV_BI_ for *A. nemorosa* and *I. glandulifera*, but had a reversed effect for *M. effusum*. We furthermore observed a reversed effect of high niche packing on ITV_BI_ for the fast colonizing non-native *I. glandulifera* (increased ITV_BI_), as compared to the slow colonizing native A. nemorosa and M. effusum (reduced ITV_BI_). Additionally, ITV_BI_ in the fast colonizing species tended to be highest for the vegetative traits plant height and leaf area, but lowest for the measured generative trait seed mass.

**Conclusions:**

This study shows that stress can both reduce and increase ITV_BI_, seemingly supporting both the stress-reduced plasticity and stress-induced variability hypotheses. Similarly, niche packing effects on ITV_BI_ supported both the niche packing hypothesis and the individual variation hypothesis. These results clearly illustrates the importance of simultaneously evaluating both abiotic and biotic factors on ITV_BI_. This study adds to the growing realization that within-population trait variation should not be ignored and can provide valuable ecological insights.

**Electronic supplementary material:**

The online version of this article (10.1186/s12898-017-0151-y) contains supplementary material, which is available to authorized users.

## Background

Functional traits determine plant species’ vital rates and fitness [1, 2]. Consequently, species under contrasting environmental conditions exhibit very different functional trait attributes due to diverging selective pressures [1, 3]. Interestingly, many plant species occur over relatively broad spatial scales and are thus exposed to strong abiotic and biotic gradients. This suggests that species exhibit large variation in their functional trait attributes across these gradients, caused by adaptation by natural selection or/and phenotypic plasticity [4]. The recent interest in intraspecific trait variation (ITV) from ecologists has detected considerable ITV in many species’ traits, even within populations (e.g. [5–9]). Since functional traits and their intraspecific variability are closely linked to plant community dynamics [10–12], ecosystem functions such as litter decomposition [13–15], ecosystem services such as disease risk reduction [15] and species responses to climate change [16–18], it is important to understand which factors drive ITV in plant species [4, 19].

Until now, most studies have focused on between-population ITV (e.g. [20–22]) and on quantifying the responsiveness of traits to environmental variation (due to trait plasticity) at the species level (e.g. [23, 24]). However, significant levels of trait variation are known to occur within populations as well (between individual ITV, ITV_BI_ sensu Albert et al. [4]) (e.g. [5, 25]). Although several studies have focused on quantifying the range of ITV_BI_ (e.g. [7, 21, 26, 27]), our understanding of what drives the extent of trait variation at the population level is still relatively limited (see for instance [17, 28–31]). Research at both the species and community level suggested that phenotypic plasticity and thus ITV_BI_ are more constrained under unfavourable abiotic conditions (environmental stress) due to environmental filtering, resulting in a decreased ITV_BI_ in cold and dry climates and in nutrient-poor sites (*stress*-*reduced plasticity hypothesis*) [29, 32, 33]. Other hypotheses state, however, that unfavourable conditions may trigger enhanced expression of phenotypic variability in traits, thus resulting in increased ITV_BI_ in stressful environments (*stress*-*induced variability hypothesis*) [8, 34, 35]. Two additional hypotheses have been proposed concerning the possible effect of biotic drivers (and more specifically of competition) on ITV_BI_. First, niche theory predicts that highly diverse plant communities should be characterized by reduced ITV_BI_, due to increased interspecific competition and selection for reduced niche overlap (communities with low niche overlap and high niche density) (*niche packing hypothesis*) [8, 36]. Similarly, asymmetrical competition for light (i.e. taller species are disproportionately advantaged) in highly productive and competitive communities is expected to have comparable effects on ITV_BI_ as high niche density (niche packing) [28, 29]. Second, the *individual variation hypothesis* assumes that all individuals in a community, irrespective of species identity, will exhibit different trait attributes to avoid (intraspecific) competition [10]. Consequently, this high within-species trait variation favours the maintenance of high species diversity, resulting in a positive correlation between ITV_BI_ and species diversity [10, 36].

Some progress has been made in quantifying the extent of community-wide trait overlap/ITV and its dependency on species richness. Support exists for both the niche packing hypothesis, with decreased ITV_BI_ in several traits, such as plant height and specific leaf area (SLA) under high species richness [8, 33] and for the individual variation hypothesis, with an increase in plant height ITV_BI_ under high species richness [37]. Although species richness provides an easily measurable proxy to quantify community-level niche density, research suggests that measures of functional diversity at the community level more readily reflect niche-based processes [38]. This suggests that using the different components of functional diversity, namely functional richness, functional evenness and functional divergence [39], likely allow a more precise assessment of the importance of niche-based processes on species-level ITV_BI_. To our knowledge, no studies have evaluated species- or functional diversity effects on ITV_BI_ patterns at the species level (however see [31, 40, 41]).

The relative importance of abiotic and biotic conditions on ITV_BI_ is furthermore expected to change along macroecological gradients. While abiotic stress is expected to increase in environmentally unfavourable conditions, competition is usually more associated with environmentally favourable conditions (cf. the stress-gradient hypothesis, [42]). Additionally, the impact of stress and competition on ITV_BI_ is most likely species- and trait-dependent. Indeed, research on invasive plant species has suggested that colonization rates (and thus invasion success) are higher for species that exhibit high ITV [43]. This suggests that species with high colonization capacity are more responsive to abiotic and biotic drivers of ITV_BI_. Regarding trait dependency, one could expect that the ITV_BI_ of growth related traits are more responsive to competition-related biotic drivers than that of reproductive traits. Similarly, the ITV_BI_ of traits that are known to be closely associated with stress responses (e.g. specific leaf area) might be more responsive to abiotic variation [3].

Here, we assess the relative effects of both abiotic (stress-related) and biotic (competition-related) variation on ITV_BI_ of four functional traits (leaf area, SLA, plant height and seed mass), for three herbaceous plant species (*Anemone nemorosa*, *Milium effusum* and *Impatiens glandulifera*) across a 2300 km long macroecological gradient in Europe. These traits form the basis of the ‘leaf-height-seed’ (LHS) plant ecology strategy scheme and have been shown to correlate with a vast number of other functional and demographic traits, and with ecosystem processes [44, 45]. We evaluated the effect of abiotic stress by quantifying climatic variables (temperature and precipitation), soil conditions (soil N, soil P and pH) and the community level stress-signature [46, 47]. The importance of biotic factors was assessed using species richness and functional diversity as proxies for niche packing, and the mean abundance weighted, community level ‘functional competition signature’ based on the C-S-R plant functional type system as a proxy for asymmetrical competition strength [46, 47].

Our three study species were specifically selected for their contrasting colonization rates, to allow the assessment of colonization rate effect on ITV_BI_ patterns. The study species were the slow colonizing *Anemone nemorosa*, a species with intermediate colonization rates, *Milium effusum*, and the fast colonizing, for the region invasive alien *Impatiens glandulifera*. Using this set-up, we aim at addressing the following research questions:

1. Does high abiotic stress result in decreased or increased ITV_BI_ for the different traits (stress-reduced plasticity vs. stress-induced variability hypothesis) resulting in either linear or quadratic relationships between ITV_BI_ and abiotic predictors? Similarly, does high biotic competition (niche density and/or asymmetrical competition) lead to decreased or increased ITV_BI_ for the different traits (niche packing vs. individual variation hypothesis) resulting in linear relationships between ITV_BI_ and biotic predictors?
2. Is the ITV_BI_ of the growth related trait plant height more affected by competition and the ITV_BI_ of the stress response related trait SLA more by abiotic factors, compared to the other traits?
3. Does the non-native, fast colonizer *I. glandulifera* exhibit larger ITV_BI_ than the slower colonizing native *A. nemorosa* and *M. effusum*, and do biotic and abiotic drivers explain a larger proportion of the variation in ITV_BI_ for *I. glandulifera* than for the *A. nemorosa* and *M. effusum*, following the expected patterns for fast colonizing species?

## Methods

### Study species

In this study we quantified ITV_BI_ for three herbaceous species across Europe, namely *A. nemorosa*, *M. effusum* and *I. glandulifera*. *Anemone nemorosa* L. (*Ranunculaceae*) is a widespread European spring ephemeral forest perennial (geophyte). The species flowers in spring, is insect-pollinated and also reproduces vegetatively through rhizomes. Individual ramets produce ten to 30 seeds per year, which are adapted to myrmecochory, resulting in a relatively low colonization rate [22, 48]. *Milium effusum* L. (*Poaceae*) is also a widespread European forest understory perennial, but differs from *A. nemorosa* by a hemicryptophytic life form, a later flowering period (early summer), adaptation to wind pollination, higher seed production (100–300 per individual per year), predominantly barochorous and epizoochorous dispersal, and a limited ability of vegetative reproduction through short stolons [17, 22, 49]. All these traits suggest an intermediate colonization rate for *M. effusum*. *Impatiens glandulifera* Royle (*Balsaminaceae*) was introduced to Europe in the 1800s from its native range in western Himalaya and subsequently became strongly invasive [50]. The species is a competitive annual of up to 2.5 m high. In its invaded range it mainly grows in riparian habitat, but has also been found to spread along road verges in more northern locations. The species produces a large number of insect-pollinated flowers in late summer, followed by up to 2500 seeds per individual per year. Reproduction is fully dependent on seed germination, with dispersal facilitated both by fruit explosion (ballistochory) and water (hydrochory) [50, 51], making the species a very fast colonizer into unoccupied habitats. All three species predominantly occur in shaded (wooded or wood-edge) habitats in Europe, which are characterized by buffered temperatures, high air humidity and high soil moisture [48–50]. For this reason, we expect these species to mainly experience stress from high temperatures and low water availability (i.e. rainfall) along the studied gradient.

### Study area

Populations were sampled in seven (*A. nemorosa* and *M. effusum*) and six (*I. glandulifera*) regions along a 2300 km macroecological gradient, ranging from Amiens, France (49.90°N, 2.30°E) in the south to Abisko, Sweden (68.35°N, 18.83°E) in the north (Table 1). In each selected region, up to six populations were randomly selected within a 20 × 20 km area, resulting in a total of 37, 39 and 34 populations for *A. nemorosa*, *M. effusum* and *I. glandulifera*, respectively (Table 1). All populations of *A. nemorosa* and *M. effusum* were sampled in 2008 and occurred in ancient deciduous forests (see [22] for more details). The populations of *I. glandulifera* were sampled in 2011 and occurred along river banks and road verges, within (recent) forests or along forest edges (see [52] for more details). No field permissions were necessary for the collection of the plant samples in this study. Plant material was formally identified by JB, SAOC, GD, AK, PDF and BJG is their respective study regions.

**Table 1:** Study region overview with average intraspecific trait variation levels

Study region (nearest city)		Amiens	Ghent	Potsdam	Bremen	Lund	Stockholm	Trondheim	Umeå	Abisko	Total
Latitude (°N)		49.9	51.1	52.4	53.1	55.7	59.3	63.4	63.8	68.4	
Longitude (°E)		2.3	3.7	13.1	8.8	13.2	18.1	10.4	20.3	18.8	
MAP (mm)		634	754	566	732	653	527	884	572	387	
GDH *A. nemorosa* (10^3^ h)		12.29	10.72	9.71	9.77	8.00	10.28	–	7.66	–	
GDH *M. effusum* (10^3^ h)		16.55	17.92	18.27	16.50	15.17	–	–	11.68	11.99	
GDH *I. glandulifera* (10^3^ h)		47.41	47.24	–	42.29	38.35	39.30	31.71	–	–	
*Anemone nemorosa*	# pops	6	5	6	6	3	6	0	5	0	37
	Plant height CV	16.8	13.5	12.4	11.6	10.4	13.4	–	14.1	–	13.5
	Seed mass CV	25.8	26.2	22.3	20.8	18.0	23.3	–	29.0	–	23.9
	SLA CV	15.0	10.9	10.3	10.4	7.6	11.8	–	11.3	–	11.4
	Leaf area CV	36.1	31.4	37.3	27.9	24.3	29.2	–	32.8	–	32.1
*Milium effusum*	# pops	5	4	6	6	6	0	0	6	6	39
	Plant height CV	22.4	18.1	14.3	14.5	12.0	–	–	10.8	11.0	14.3
	Seed mass CV	23.6	20.9	26.8	16.9	17.6	–	–	19.9	22.7	21.2
	SLA CV	11.6	9.3	12.3	7.3	9.2	–	–	13.0	20.2	12.0
	Leaf area CV	30.6	27.7	27.6	22.2	21.9	–	–	21.9	33.3	26.3
*Impatiens glandulifera*	# pops	6	6	0	5	6	5	6	0	0	34
	Plant height CV	19.5	23.0	–	15.3	15.6	19.2	22.9	–	–	19.4
	Seed mass CV	19.5	23.1	–	17.9	19.3	18.6	18.7	–	–	19.6
	Leaf area CV	23.0	38.3	–	26.4	30.8	42.8	51.1	–	–	36.7

Location, climatic characterization, number of sampled populations (# pops) and mean population level functional trait coefficients of variation (CV) within each study region

*GDH* growing degree hours, *MAP* long-term mean annual precipitation, *SLA* specific leaf area

### Functional traits of the study species

Leaf area, plant height and seed mass were measured for all three studied species, while specific leaf area (SLA) was not measured for *I. glandulifera*. For *A. nemorosa* and *M. effusum*, plant height was measured and all seeds and leaves of 15 randomly selected individual plants per population were collected within a 25 × 25 m area for both species at seed maturity. Seed mass was defined as the mean air-dry seed mass per seed of all seeds per individual for *A. nemorosa* and mean air-dry seed mass per seed of 50 randomly selected seeds per individual for *M. effusum*. Plant height, seed mass and SLA measurements were performed according to standardized protocols [22, 53]. Leaf area of the flattened leaves was measured using a Li-Cor Portable Area Meter Li-3000 (Li-Cor Biosciences, NE, USA) after 72 h air-drying (50 °C) [22]. SLA was hence calculated using dry leaf area due to logistic constraints. We nonetheless expect this ‘shrinkage bias’ to be constant across individuals within a species, hence justifying its use for ITV comparisons [22].

For *I. glandulifera*, plant height was measured and ten capsules (fruits) were collected for 30 randomly selected individual plants per population within a 25 × 25 m area. Seed mass was calculated as the mean dry seed mass of all seeds of ten capsules per individual. Leaf area of *I. glandulifera* was estimated as the product of leaf length and leaf width of the largest leaf of each individual plant. This has previously been shown to be a reasonable proxy for leaf area [17]. In all species, only healthy, non-damaged individuals were selected for functional trait measurements. Within-population functional trait variation (ITV_BI_) was subsequently quantified by calculating both the standard deviation (SD) and the (dimensionless) coefficient of variation (CV) for each trait per population [7, 17].

### Abiotic variables

Mean annual precipitation (MAP) for the period 1982–2002 was retrieved from the closest city (all < 40 km from the sampled populations) to each sampling region from the Climate-Data.org model [54] (cities given in Table 1) (cf. [55]). As a temperature proxy we used the number of growing degree hours (GDH) above 5 °C between 1st January and the day of trait collection, rather than mean annual temperature, since GDH is considered to be more tightly related to plant development [55]. GDH was calculated as defined in Lindsey and Newman [56] based on observed daily minimum and maximum temperatures during the sampling year, which was 2008 for *A. nemorosa* and *M. effusum* and 2011 for *I. glandulifera*, obtained for the closest city to each sampling region from NOAA [57].

For each *A. nemorosa* and *M. effusum* population, five 4-cm deep soil cores were collected and pooled to measure soil pH (determined from a solution of 10 g of soil and 25 ml of 0.01 M CaCl_2_ with a standard glass electrode), plant available phosphorus (P) (extraction with ammonium lactate and photometric determination by flow injection analysis and flame atomic absorption spectrophotometry, SpectrAA-220, Varian) and total soil nitrogen (N) (elemental analyzer; %) [22]. For each *I. glandulifera* population, plant available soil nitrogen (nitrate and ammonium), rather than total soil N was measured during 6 weeks of the growing season (mid-June until the end of July 2011) using four anion and cation plant root simulator (PRS) soil probes (Western Ag Innovations Inc., Saskatoon, Canada). After retrieval from the field, the probes were shipped to the analytical laboratory of Western Ag Innovations for analysis. There, inorganic nitrogen levels (NH_4_^+^ and NO_3_^−^) were quantified by colorimetry using a Technicon Autoanalyzer II. The NO_3_^−^ analysis was slightly modified through the addition of NaOH to the NH_4_Cl reagent in order to neutralize the sample solution before its entry into the Cd-reduction column (Western Ag Labs 2003).

### Biotic variables

Community composition was determined for each population of the three study species by recording the cover-abundance (% cover) of all (understory) vascular plant species present within the 25 × 25 m area. For functional diversity calculations, we selected ten functional plant traits related to species growth, survival and reproduction, namely plant height, life span, rosette type, leaf area, specific leaf area, leaf dry matter content, age of first flowering, reproductive type, seed number and seed mass (Additional file 1). Trait attributes for all co-occurring species were extracted from several online databases, with an average data availability of 92% (Additional file 1) [58, 59]. Plant height, leaf area, seed number and seed mass of co-occurring species were logarithmically transformed to reduce the influence of extreme values in subsequent functional diversity calculations.

The resulting plot × species (111 × 167) and species × trait (167 × 10) matrices were used to calculate species richness and three measures of functional diversity for the plant communities present at each population of the three study species, namely functional richness, functional evenness and functional divergence [60] with the ‘FD’ package in R version 3.2.4 [61]. These measures addressed the three independent components of functional diversity, respectively; the amount of filled trait space, the regularity of the distribution of abundance in the trait space, and the spread or divergence in the distribution of abundance within the trait space [39]. Note that for each given study site, we did not include the respective study species (*A. nemorosa*, *M. effusum* or *I. glandulifera*), nor its functional trait attributes in the species richness and functional diversity calculations. Prior to functional diversity calculations, all traits were z-transformed and missing trait values were replaced using multivariate imputation with chain equations (MICE [62]), using the ‘mice’ R package [63]. This method uses predictive mean matching with five imputations, with the average of these five imputed value used to replace the missing value and has been shown to produce superior results compared to other imputation methods for database plant traits [64].

Additionally, we calculated the mean abundance weighted ‘functional competition signature’ (C-sign.) and ‘functional stress signature’ (S-sign.) based on the C-S-R plant functional type system [46], following the method of Hunt et al. [47]. The C-S-R functional type of each species was collected from Hunt et al. [47] and the BiolFlor database [58]. Since this C-signature is based on the present species communities, we believe that they are adequate indicators of the overall community-level strength of the asymmetrical competition for resources such as light at these locations. Similarly, we believe that the S-signature presents a proxy of the extent of (abiotic) stress experienced by each community. The full dataset is included in Additional file 2.

### Statistical analysis

First, we performed Spearman rank correlations between latitude on the one hand, and all abiotic (MAP, GDH, soil N, soil P, pH, S-sign.) and biotic predictor (species richness, C-sign. and functional richness, evenness and divergence) on the other hand. Secondly, we explored the correlation between both measures of ITV_BI_ (SD and CV) on the one hand, and population-level mean trait values, on the other hand, using linear mixed-effect models with study region as a random intercept factor. This random intercept was included to account for the dependence of populations within each region. As expected, SD was strongly correlated with trait means for most traits, justifying the use of CV as a trait mean-independent proxy for ITV_BI_ (Additional file 3). Next, we assessed correlations between the ITV_BI_ measures of all traits, for each study species separately, using Spearman rank correlations.

We assessed the effects of the measured abiotic and biotic factors along the gradient on between individual functional trait variation per population with linear mixed-effect models. Note that for these models we used trait CV as a measure of ITV_BI_ for all traits. An individual mixed-effect model was constructed for each population level functional ITV_BI_ as response variable, for each study species separately, with study region as a random intercept factor and all abiotic, stress-related (MAP, GDH, soil N, soil P, pH, S-sign.) and biotic/competition-related (species richness, C-sign. and functional richness, evenness and divergence) predictors as fixed effects (predictors). Quadratic terms for the stress-related predictors (MAP, GDH, soil N, soil P, pH, S-sign.) were also included as fixed effects. Since the optimal growing conditions (lowest stress) can potentially occur at intermediate levels of the assessed abiotic variables, quadratic relationships could be expected under both the stress-reduced plasticity hypothesis (convex parabolic relation) and stress-induced variability hypothesis (concave parabolic relation). Note that quadratic relationships are not expected for the biotic predictors since competition is expected to vary unidirectionally with the assessed competition-related biotic predictors.

Models were run after scaling (standardizing) all variables, as such allowing parameter (beta coefficient) comparisons. Final models were obtained using stepwise model reduction based on the Akaike’s information criterion (AIC) for models based on ML estimations (Table 1). All final model output was based on REML estimations. Marginal and conditional R^2^-coefficients, respectively describing the proportion of variance explained by the fixed factors and the variance explained by both the fixed and random factors, were calculated for each final model (cf. [65]). Several response variables and predictors were transformed (prior to standardization) to satisfy both the normal distribution of residuals and independence of residuals model assumptions (Table 2). Before the linear mixed-effect model analyses, all predictors were checked for multicollinearity using variation inflation factors (VIF) for each study species separately, but no collinearity problems were observed with a conservative VIF threshold of five. All linear mixed-effect models were performed with the ‘nlme’ package [66] in R 3.3.3., all other statistical analyses were performed in SPSS 21.0 (SPSS Inc., Chicago, IL, US).

**Table 2:** Parameter estimates of the performed reduced linear mixed-effect models for each functional trait ITV_BI_ measure separately

Species	Functional trait CV	${\text{R}}_{\text{M}}^{ 2} /{\text{R}}_{\text{C}}^{ 2}$	AIC/ΔAIC	Climate	Local abiotic	Local biotic
*Anemone nemorosa*, (N = 37)	Plant height	0.524/0.531	90.86/10.29	GDH 12.75*/− 5.73	Soil N^b^ 7.29*/0.42	Species richness 6.26*/− 0.31
				GDH^2^ 14.70*/6.25		Functional divergence 7.41*/− 0.41
						C-sign. 9.59**/0.46
	Seed mass^b^	0.202/0.202	105.18/12.22	–	–	Functional evenness^c^ 4.43*/− 0.32
						C-sign. 4.86*/0.34
	SLA^a^	0.442/0.442	92.45/3.49	MAP 4.57*/− 0.30	S-sign. 8.76**/0.43	Functional richness 3.09^(^*^)^/− 0.24
						Functional evenness^c^ 7.77**/− 0.37
	Leaf area	< 0.001/0.243	102.28/2.18	–	–	–
*Milium effusum*, (N = 39)	Plant height	0.236/0.434	105.37/16.17	GDH 5.31^(^*^)^/0.51	–	–
	Seed mass	0.362/0.362	105.98/5.44	MAP 9.09*/− 0.56	Soil N 8.57**/− 1.53	–
				GDH 12.70*/0.63	Soil N^2^ 7.09*/1.44	
	SLA^a^	0.803/0.803	58.93/18.68	MAP 41.50***/− 0.57	Soil N 14.68***/− 1.25	Functional divergence 5.26*/− 0.18
					Soil N^2^ 19.30***/1.50	
					S-sign. 10.47**/− 0.27	
					S-sign.^2^ 10.49**/0.25	
	Leaf area	< 0.001/0.272	112.11/8.82	–	–	–
*Impatiens glandulifera*, (N = 34)	Plant height	0.445/0.445	88.86/4.33	MAP 14.66*/− 8.85	S-sign. 9.18**/− 0.48	Functional evenness 5.38*/0.38
				MAP^2^ 15.54*/9.31		
				GDH 5.48*/0.50		
	Seed mass^b^	0.110/0.110	99.41/6.43	–	–	Functional evenness 4.06^(^*^)^/0.34
	Leaf area^b^	0.580/0.580	80.54/4.70	MAP 19.86*/− 6.70	S-sign. 6.39*/− 0.32	Functional divergence 3.49^(^*^)^/0.24
				MAP^2^ 21.78*/7.01		

Marginal (${\text{R}}_{\text{M}}^{ 2}$), conditional R^2^ (${\text{R}}_{\text{C}}^{ 2}$), AIC of the best model and ΔAIC for the full (initial) model given for each final model. Test statistic (F) and P-value (before slash) and beta-coefficient (after slash) given for each retained predictor after model reduction. All models based on coefficient of variation (CV)

*C-sign.* mean abundance weighted functional competition signature, *GDH* growing degree hours, *MAP* mean annual precipitation, *S-sign.* mean abundance weighted functional stress signature, *SLA* specific leaf area, *soil N* soil nitrogen concentration

(*) 0.10 ≥ P-value > 0.05; * 0.05 ≥ P-value > 0.01; ** 0.01 ≥ P-value > 0.001; *** 0.001 ≥ P-value

^a^Square root transformation, ^b^ logarithm transformation, ^c^ squared transformation

## Results

The extent of between individual intraspecific trait variation (ITV_BI_) was variable across traits, with on average the lowest variability for SLA [across species mean CV = 11.7 ± 4.0% (SD)], followed by plant height (mean CV = 15.6 ± 5.3%) and seed mass (mean CV = 21.6 ± 6.1%), and the highest variability for leaf area (mean CV = 31.5 ± 9.7%). Interestingly, the relative ranking of trait variabilities (CV) was consistent across all three study species (Table 1). When comparing the ITV_BI_ (CV) for the different traits across species, it was not consistently the same study species exhibiting the highest ITV_BI_, with the highest mean variability in plant height and leaf area for *I. glandulifera*, the highest mean variability in SLA for *M. effusum* and the highest mean variability in seed mass for *A. nemorosa* (Table 1). ITV_BI_ was positively correlated among all traits for *A. nemorosa*, except between SLA and leaf area and between SLA and seed mass. For *M. effusum* positive correlations only occurred between seed mass, SLA and leaf area ITV_BI_, while for *I. glandulifera*, only leaf area ITV_BI_ correlated positively with all other traits (Additional file 4).

Several abiotic and biotic variables were significantly related to latitude. Both temperature and precipitation decreased with latitude, while species richness (for *M. effusum*), functional diversity (for *A. nemorosa* and *M. effusum*) and the functional competition signature (for *M. effusum*) increased with latitude (Additional file 5). Soil variables were mainly unrelated to latitude, with only for *A. nemorosa* a significant increase in soil nitrogen with latitude (Additional file 5).

**Fig. 1:**
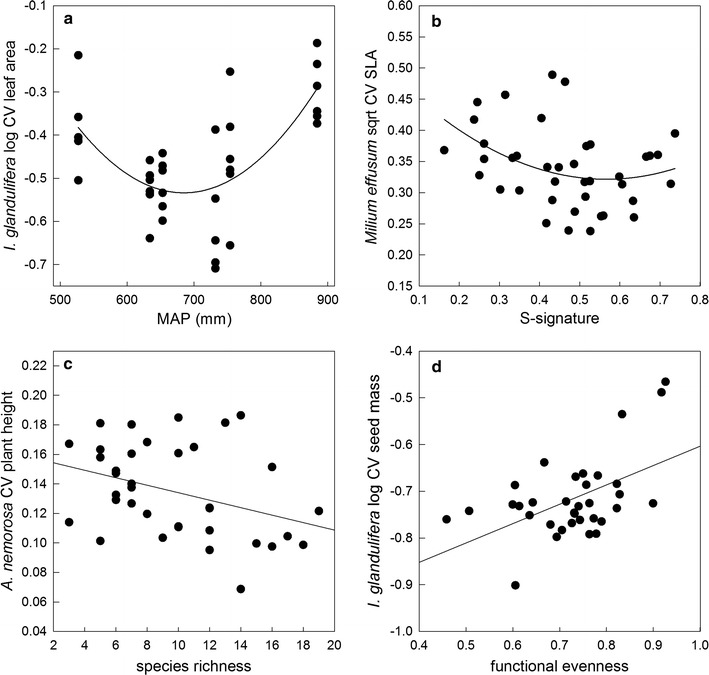
Relationships between intraspecific trait variation (cf. ITV_BI_) and several abiotic and biotic factors. **a** Relationship between mean annual precipitation (MAP) and the log-transformed coefficient of variation (CV) of leaf area for *Impatiens glandulifera*, **b** relationship between stress-signature and the square-root-transformed CV of specific leaf area (SLA) for *Milium effusum*, **c** relationship between species richness and the CV of plant height for *Anemone nemorosa*, **d** relationship between functional evenness and the log-transformed CV of seed mass for *Impatiens glandulifera*. Regression lines present statistically significant linear relationships

Climatic variables significantly affected ITV_BI_. Mean annual precipitation (MAP) during 1982–2002 was negatively correlated with the CV of SLA of *A. nemorosa* and the CV of seed mass and SLA of *M. effusum*. MAP furthermore showed a convex parabolic relation with the CV’s of both plant height and leaf area of *I. glandulifera* (Table 2, Fig. 1a). Temperature (Growing degree hours, GDH) was positively correlated with the CV’s of plant height and seed mass of *M. effusum* and the CV of plant height of *I. glandulifera*. GDH also showed a convex parabolic relation with the CV of plant height of *A. nemorosa* (Table 2). Concerning the effects of the soil, we observed a positive correlation between soil nitrogen and the CV of plant height of *A. nemorosa* and a convex parabolic relation with the CV’s of both seed mass and SLA of *M. effusum*. We did also observe a clear response to increased stress levels, as quantified through the S-signature, with a positive correlation with the CV of SLA of *A. nemorosa*, a negative correlation with the CV’s of both plant height and leaf area of *I. glandulifera* and a convex parabolic relation with the CV of SLA of *M. effusum* (Table 2, Fig. 1b).

Several biotic factors also affected ITV_BI_. First, plant height variability in *A. nemorosa* was directly negatively affected by the increased niche density associated with increased species richness (significant species richness effect, Fig. 1c), while *A. nemorosa*’s SLA variability was negatively affected by increasing functional richness (Table 2). Second, functional evenness was negatively correlated with the CV’s of seed mass and SLA of *A. nemorosa* and positively correlated with the CV’s of both plant height and seed mass of *I. glandulifera* (Table 2, Fig. 1d). Functional divergence was furthermore negatively related to the CV of plant height of *A. nemorosa* and the CV of SLA of *M. effusum* and positively related to the CV of leaf area of *I. glandulifera* (Table 2). Finally, the proxy for vegetation-level asymmetrical competition strength (C-signature) was positively related to variation in both plant height and seed mass for *A. nemorosa* (Table 2).

## Discussion

### Stress and competition effects

Although responses tended to be mainly species-specific, we observed both decreased (*stress*-*reduced plasticity hypothesis*, [32]) and increased (*stress*-*induced variability hypothesis*, [34]) ITV_BI_ under (abiotically) stressful conditions, although climatic stress (high temperature and low precipitation) nevertheless consistently increased ITV_BI_. Similarly, we observed both reduced (*niche packing hypothesis*, [36]) and enhanced (*individual variation hypothesis*, [10]) ITV_BI_ under high community-level niche density.

Interestingly, both drought stress (low precipitation) and (niche) competition (functional diversity, functional competition signature) increased with latitude, although heat stress (high GDH) decreased with latitude. This was unexpected, since theory predicts competition and abiotic stress to trade-off across latitudes [33, 42]. This suggests that these plants are occurring in milder microclimates in climatically harsher locations, as such allowing them to circumvent more extreme macroclimatic stress conditions. This likely results in the occurrence of these species in more competitive, mesic communities in desirable, mild microsites in the harsher (drier) north [67]. This could furthermore explain the absence of a clear correlation between the biotically inferred mean community-level stress level (S-sign.) and climatic factors. These results further emphasize the importance of assessing both abiotic and biotic factors along macroecological gradients to get a full picture of potential drivers of ITV_BI_, since both are not always predictably related [30, 33].

Climate (temperature and precipitation) significantly affected ITV_BI_ for several traits in each study species. The direction of these correlations were furthermore consistent across species and traits, with either reduction of trait variation at low temperatures (GDH) and high precipitation (MAP), or trait variation reduction at intermediate temperatures/precipitation levels (climatically favourable locations). These results strongly suggest the prevalence of the stress-induced variability hypothesis. Indeed, considering that these species predominantly occur in relatively cool (shaded) locations with high humidity and soil moisture [48–50], we expect climatic stress to be strongest at high GDH and low MAP levels. Although note that the observed convex parabolic relationships indicate that for *A. nemorosa* and *I. glandulifera* the climatically favourable locations seem to occur at intermediate levels of GDH and MAP, respectively. One soil variable (nitrogen) had a similar effect for several traits of *M. effusum*, with a reduction in ITV_BI_ at intermediate soil nitrogen levels, again indicative of the stress-induced variability hypothesis. This pattern was also mirrored in the biotically inferred stress parameter (S-sign.), which is usually indicative of soil nutrient (stress) levels. For *A. nemorosa* and *I. glandulifera* however, soil nutrient-related stress seemed to mainly support the stress-reduced plasticity hypothesis, with lowest ITV_BI_ at low soil nitrogen levels or high S-sign. levels, These results are largely in agreement with previous studies of nutrient effects on ITV_BI_ [17, 29]. Overall, our results support the recent findings of inconsistent effects of environmental stress on ITV_BI_ across traits and species [17, 30], and suggest that climatic factors and nutrient stress can differently affect ITV_BI_ levels.

Concerning the proxies for niche-based effects on ITV_BI_, we can assume that communities with high species/functional richness and high functional evenness have high niche density. High functional divergence is usually expected to be a result of strong niche filtering during community assembly, and thus indicates the presence of strong niche-based effects within the community [68]. Our results show that for *A. nemorosa* and *M. effusum*, ITV_BI_ is consistently reduced under high niche density. This is in agreement with the results of the meta-analysis of Siefert et al. [8], which looked at the effect of species richness on community-level ITV_BI_. The ITV_BI_ of *I. glandulifera*, however, is enhanced under the same conditions, a pattern that has previously also been observed at the community-level for limestone grasslands on Öland (Sweden) [37]. This suggests that the niche packing hypothesis seemingly drives trait variation in the native *A. nemorosa* and *M. effusum* (cf. [8, 33, 36]), but that the individual variation hypothesis applies to the ITV_BI_ of the invasive *I. glandulifera* (cf. [10, 36]). Additionally, community-level asymmetrical competition strength (expressed by the C-signature) led to increased ITV_BI_ for two traits in *A. nemorosa*. Hence, contrary to our expectations, asymmetrical competition may trigger increased trait variation [28], while niche density reduced this variation for the same traits in the same species. Interestingly, Burns and Strauss [40] observed a similar increase in trait variation under increased interspecific (hierarchical) competition in an experimental setup. It has been argued that this trait variation may be an adaptive response to asymmetrical competition [17, 40].

We did not find clear support for our hypotheses that ITV_BI_ of growth related traits, such as plant height, are more related to competition, or that ITV_BI_ in stress-related traits, such as SLA, is best explained by abiotic variables. Indeed, ITV_BI_ in both traits was affected by a combination of abiotic and biotic drivers. These patterns might be the result of linkage between several functional traits, rendering them non-independent (Additional file 4) [44]. Additionally, neither plant height nor SLA are likely to be solely related to either stress-related abiotic or competition-related biotic responses. Indeed, although plant height is often linked to competition strategies in plants, both light- and nutrient stress are known to affect plant height as well [8, 44]. Similarly, although SLA is often linked to stress responses in plants, it is also known to be affected by reduced light levels due to competition effects [3, 44].

### Colonization rate

We partly found support for the expected higher ITV_BI_ in species with faster colonization rates [43]: overall ITV_BI_ (CV) values for the vegetative traits plant height and leaf area in the fast colonizing, invasive *I. glandulifera* were higher than those in the slow colonizing *A. nemorosa* and intermediate colonizer *M. effusum*. Unexpectedly, leaf area ITV_BI_ was lowest for *M. effusum*, and not for *A. nemorosa*. This pattern of higher ITV_BI_ for the fast colonizer was only apparent for vegetative traits, however, with a reversed pattern for the measured reproductive trait (seed mass). Since both *A. nemorosa* and *M. effusum* occur in relatively stable ancient forests, these slowly colonizing species have most likely experienced consistent, long term environmental selection on their functional trait set, thus resulting in more optimal mean trait attributes and reduced ITV_BI_ [17, 22]. The invasive *I. glandulifera*, on the other hand, mainly occurs in more disturbed recent forests and forest edges, where these young, dynamic populations have been exposed to shorter periods of much less consistent, fluctuating environmental selection, thus explaining the occurrence of higher ITV_BI_ [50, 51]. Alternatively, species with greater dispersal ability are likely exposed to a greater range of environmental variability, possibly leading to selection for increased phenotypic plasticity [43]. These effects are likely aggravated by differences in plant height between the three species. Indeed, while both *A. nemorosa* and *M. effusum* are short understory species buffered against environmental fluctuation by larger understory species, *I. glandulifera* is not buffered by these effects, since it usually is the dominant ‘canopy species’ of the forest understory. The reduced ITV_BI_ variation in seed mass for *I. glandulifera* might be the result of stronger selection on seed traits for this species, compared to the partially clonal, perennial *A. nemorosa* and *M. effusum*. Indeed, since this species is annual, population persistence is fully dependent on annual seedling establishment.

There was no consistently stronger effect of either abiotic or biotic drivers on trait ITV_BI_ of the fast colonizing *I. glandulifera*. We rather observed species-specific responses of the different abiotic and biotic drivers, making overall generalizations difficult. However, as previously mentioned, the native, slower colonizing species *A. nemorosa* and *M. effusum* were characterized by a reduction in ITV_BI_ under increased niche density, as opposed to the exotic, fast colonizing *I. glandulifera*, which showed an increase in trait variation under high niche density. Such a potential for large trait variation under high competition intensity, through either plastic responses or genetic adaptation, might explain the invasive success of the non-native *I. glandulifera* in Europe [43].

## Conclusions

We found clear effects of both stress-related abiotic drivers and competition-related biotic drivers on within-population intraspecific functional trait variation. Our results suggest that stress can both reduce and increase ITV_BI_, seemingly supporting both the stress-reduced plasticity and stress-induced variability hypotheses (cf. [32, 34]). Climatic stress consistently increased ITV_BI_ across species and traits. Soil nutrient stress, on the other hand, reduced ITV_BI_ for *A. nemorosa* and *I. glandulifera*, but not for *M. effusum*. Similarly, niche packing effects on ITV_BI_ were both negative and positive, respectively supporting the niche packing hypothesis [36] and the individual variation hypothesis [10]. This clearly illustrates the importance of simultaneously evaluating both abiotic and biotic factors on ITV_BI_ [30, 33].

In sum, ITV_BI_ for several (vegetative) traits tended to be highest in the non-native, fast colonizing species. Second, we observed a reversed effect of niche density (functional diversity) on ITV_BI_ for the fast colonizing invasive *Impatiens glandulifera*, as compared to the slow(er) colonizing native *Anemone nemorosa* and *Milium effusum*. Finally, our study adds to the growing realization that within-population trait variation should not be ignored in ecological studies and can even provide valuable ecological insights [4, 8, 12, 19, 22, 25].

## Additional files

**Additional file 1.** Overview of the selected functional traits for the functional diversity analysis. Description, scale, percentage of available data for all species and main data sources are given for each trait.

**Additional file 2.** The full dataset used for this study. Study species, study region, population code, all trait SD’s and CV’s, latitude and all abiotic and biotic predictors given for each sampled population of *Anemone nemorosa*, Milium effusum and Impatiens glandulifera. C-signature = mean abundance weighted functional competition signature, CV = coefficient of variation, GDH = growing degree hours, MAP = mean annual precipitation, NA = not available, S-signature = mean abundance weighted functional stress signature, SD = standard deviation, soil N = soil nitrogen, soil P = soil phosphorous.

**Additional file 3.** Parameter estimates for the separate linear mixed-effect models between each trait ITV_BI_ (SD and CV) (response) and population-level trait mean (predictor). Test statistic (F) and *P* value (before slash), and beta-coefficient (after slash) given for each model. CV = coefficient of variation, SLA = specific leaf area, SD = standard deviation. Significance: ^(*)^: 0.10 ≥ P-value > 0.05 ^*^: 0.05 ≥ P-value > 0.01; ^**^: 0.01 ≥ P-value > 0.001; ^***^: 0.001 ≥ P-value. ^a^ = predictor square root transformed, ^b^ = predictor logarithmic transformed, ^c^ = response square root transformed, ^d^ = response logarithmic transformed.

**Additional file 4.** Pairwise spearman rank correlations between all trait ITV_BI_, for each study species separately. ITV_BI_ is quantified as coefficient of variation (CV) for all traits. Spearman rank correlation coefficients given for each test. SLA = specific leaf area. Significance: ^(*)^: 0.10 ≥ P-value > 0.05 ^*^: 0.05 ≥ P-value > 0.01; ^**^: 0.01 ≥ P-value > 0.001; ^***^: 0.001 ≥ P-value.

**Additional file 5.** Spearman rank correlations between latitude and the different climatic, soil and local biotic variables, for each study species separately. Spearman rank correlation coefficients given for each test. C-sign. = mean abundance weighted functional competition signature, GDH = growing degree hours, MAP = mean annual precipitation, S-sign. = mean abundance weighted functional stress signature, soil N = soil nitrogen, soil P = soil phosphorous. Significance: ^(*)^: 0.10 ≥ P-value > 0.05 ^*^: 0.05 ≥ P-value > 0.01; ^**^: 0.01 ≥ P-value > 0.001; ^***^: 0.001 ≥ P-value.

## Abbreviations

C-sign.: functional competition signature
CV: coefficient of variation
GDH: growing degree hours
ITV: intraspecific trait variation
ITV_BI_: between individual intraspecific trait variation
MAP: mean annual precipitation
S-sign.: functional stress signature
SD: standard deviation
SLA: specific leaf area
soil N: soil nitrogen
soil P: soil phosphorous
