# Supplementary material for: Biotic and abiotic drivers of intraspecific trait variation within plant populations of three herbaceous plant species along a latitudinal gradient
Source: BMC Ecol. 2017 Dec 12;17:38. doi: 10.1186/s12898-017-0151-y (PMC5727960; doi:10.1186/s12898-017-0151-y)
Supplement: Supplementary file 1 — Additional file 1. Overview of the selected functional traits for the functional diversity analysis. Description, scale, percentage of available data for all species and main data sources are given for each trait. [file 12898_2017_151_MOESM1_ESM.pdf]

Additional file 1. Overview of the selected functional traits for the functional diversity analysis.

trait	description	scale	% of data available	data source
plant height	mean individual height (m), logarithmically transformed	ratio	98.2	Kleyer et al. 2008
life span	1. annual; 2. biennial; 3. once flowering perennial; 4. multiple flowering perennial	ordinal	98.2	Klotz et al. 2002
rosette type	1. rosette; 2. half-rosette; 3. no rosette	ordinal	98.2	Klotz et al. 2002
leaf area	mean leaf area (mm^2), logarithmically transformed	ratio	94.0	Kleyer et al. 2008
SLA	specific leaf area (mm^2/mg), leaf fresh size per leaf dry mass	ratio	97.6	Kleyer et al. 2008
LDMC	leaf dry matter content (mg/g) , leaf dry mass per leaf fresh mass	ratio	95.8	Kleyer et al. 2008
age of first flowering	1. < 1 year; 2. between 1 and 5 years; 3. > 5 years	ordinal	71.3	Kleyer et al. 2008
reproductive type	1. mainly seeds/spores; 2. seeds/spores & vegetative; 3. mainly vegetative	ordinal	98.2	Klotz et al. 2002
seed number	number of seeds per plant, logarithmically transformed	ratio	88.6	Kleyer et al. 2008
seed mass	mass of a single seed (mg), logarithmically transformed	ratio	80.8	Kleyer et al. 2008

Description, scale, percentage of available data for all species and main data sources are given for each trait.
