# Supplementary material for: Biotic and abiotic drivers of intraspecific trait variation within plant populations of three herbaceous plant species along a latitudinal gradient
Source: BMC Ecol. 2017 Dec 12;17:38. doi: 10.1186/s12898-017-0151-y (PMC5727960; doi:10.1186/s12898-017-0151-y)
Supplement: Supplementary file 3 — Additional file 3. Parameter estimates for the separate linear mixed-effect models between each trait ITVBI (SD and CV) (response) and population-level trait mean (predictor). Test statistic (F) and P value (before slash), and beta-coefficient (after slash) given for each model. CV = coefficient of variation, SLA = specific leaf area, SD = standard deviation. Significance: (*): 0.10 ≥ P-value > 0.05 *: 0.05 ≥ P-value > 0.01; **: 0.01 ≥ P-value > 0.001; ***: 0.001 ≥ P-value. a = predictor square root transformed, b = predictor logarithmic transformed, c = response square root transformed, d = response logarithmic transformed. [file 12898_2017_151_MOESM3_ESM.pdf]

Additional file 3. Parameter estimates for the separate linear mixed-effect models between each trait ITV_{BI} (SD and CV) (response) and population-level trait mean (predictor).

	Anemone nemorosa (N = 37)		*Milium effusum* (N = 39)		*Impatiens glandulifera* (N = 34)	
	**SD**	**CV**	**SD**	**CV**	**SD**	**CV**
plant height	$12.95^{**}/0.575^{ac}$	$0.22/-0.092^{a}$	$<0.01/0.008^{ac}$	$13.07^{**}/-0.603^{ad}$	$23.97^{***}/0.700$	0.35/0.097
seed mass	$0.24/-0.090^{d}$	$17.39^{**}/-0.683^{c}$	$4.46^{*}/0.341^{bd}$	$2.17/-0.262^{b}$	$27.11^{***}/0.736^{b}$	$0.56/0.134^{bd}$
SLA	$3.91^{*}/0.379^{d}$	$2.65/-0.317^{c}$	$3.95^{*}/0.300$	$3.56^{(*)}/-0.275^{c}$	-	-
leaf area	$72.12^{***}/0.854^{bd}$	$1.49/0.220^{b}$	$23.43^{***}/0.679$	<0.01/0.006	$56.62^{***}/0.874$	$9.62/-0.483^{d}$

Test statistic (F) and P-value (before slash), and beta-coefficient (after slash) given for each model. CV = coefficient of variation, SLA = specific leaf area, SD = standard deviation. Significance: $^{(*)}$: 0.10 ≥ P-value > 0.05 *: 0.05 ≥ P-value > 0.01; **: 0.01 ≥ P-value > 0.001; ***: 0.001 ≥ P-value. ^{a} = predictor square root transformed, ^{b} = predictor logarithmic transformed, ^{c} = response square root transformed, ^{d} = response logarithmic transformed.
