# Supplementary material for: Biotic and abiotic drivers of intraspecific trait variation within plant populations of three herbaceous plant species along a latitudinal gradient
Source: BMC Ecol. 2017 Dec 12;17:38. doi: 10.1186/s12898-017-0151-y (PMC5727960; doi:10.1186/s12898-017-0151-y)
Supplement: Supplementary file 4 — Additional file 4. Pairwise spearman rank correlations between all trait ITVBI, for each study species separately. ITVBI is quantified as coefficient of variation (CV) for all traits. Spearman rank correlation coefficients given for each test. SLA = specific leaf area. Significance: (*): 0.10 ≥ P-value > 0.05 *: 0.05 ≥ P-value > 0.01; **: 0.01 ≥ P-value > 0.001; ***: 0.001 ≥ P-value. [file 12898_2017_151_MOESM4_ESM.pdf]

Additional file 4. Pairwise spearman rank correlations between all trait ITV_{BI}, for each study species separately.

		A. nemorosa (N = 37)	***M. effusum*** (N = 39)	***I. glandulifera*** (N = 34)
plant height	seed mass	0.383^{*}	0.106	0.222
plant height	SLA	$0.276^{(*)}$	-0.199	-
plant height	leaf area	0.433^{**}	-0.100	0.729^{***}
seed mass	SLA	0.201	$0.313^{(*)}$	-
seed mass	leaf area	0.318^{*}	0.325^{*}	$0.294^{(*)}$
SLA	leaf area	0.181	0.475^{**}	-

ITV_{BI} is quantified as coefficient of variation (CV) for all traits. Spearman rank correlation coefficients given for each test. SLA = specific leaf area. Significance: $^{(*)}$: 0.10 ≥ P-value > 0.05 *: 0.05 ≥ P-value > 0.01; **: 0.01 ≥ P-value > 0.001; ***: 0.001 ≥ P-value.
