# Supplementary material for: Biotic and abiotic drivers of intraspecific trait variation within plant populations of three herbaceous plant species along a latitudinal gradient
Source: BMC Ecol. 2017 Dec 12;17:38. doi: 10.1186/s12898-017-0151-y (PMC5727960; doi:10.1186/s12898-017-0151-y)
Supplement: Supplementary file 5 — Additional file 5. Spearman rank correlations between latitude and the different climatic, soil and local biotic variables, for each study species separately. Spearman rank correlation coefficients given for each test. C-sign. = mean abundance weighted functional competition signature, GDH = growing degree hours, MAP = mean annual precipitation, S-sign. = mean abundance weighted functional stress signature, soil N = soil nitrogen, soil P = soil phosphorous. Significance: (*): 0.10 ≥ P-value > 0.05 *: 0.05 ≥ P-value > 0.01; **: 0.01 ≥ P-value > 0.001; ***: 0.001 ≥ P-value. [file 12898_2017_151_MOESM5_ESM.pdf]

Additional file 5. Spearman rank correlations between latitude and the different climatic, soil and local biotic variables, for each study species separately.

	A. nemorosa (N = 37)	*M. effusum* (N = 39)	*I. glandulifera* (N = 34)
**climate**			
MAP	-0.431**	-0.484**	0.235
GDH	-0.719***	-0.853***	-0.871***
**soil**			
soil N	0.532**	0.154	-0.096
soil P	0.282(*)	0.095	-
pH	-0.207	0.291(*)	-
S-sign.	-0.321(*)	-0.246	0.205
**local biotic**			
Species richness	0.150	0.419**	0.200
functional richness	0.091	0.073	-0.007
functional evenness	0.484**	0.07	-0.263
functional divergence	0.369*	0.415*	0.124
C-sign.	-0.044	0.274(*)	-0.044

Spearman rank correlation coefficients given for each test. C-sign. = mean abundance weighted functional competition signature, GDH = growing degree hours, MAP = mean annual precipitation, S-sign. = mean abundance weighted functional stress signature, soil N = soil nitrogen, soil P = soil phosphorous. Significance: (*): 0.10 ≥ P-value > 0.05 *: 0.05 ≥ P-value > 0.01; **: 0.01 ≥ P-value > 0.001; ***: 0.001 ≥ P-value.
